# Problems in Implication of Epidemiology Submitted as Evidence to the Court of Air Pollution Health Damages Trials

**DOI:** 10.2188/jea.11.276

**Published:** 2007-11-30

**Authors:** Kazuho Maeda

**Affiliations:** Professor Emeritus, University of Tokyo.

**Keywords:** air pollution health effects, civil lawsuit, difference between judicial and scientific interpretation, epidemiology

## Abstract

Civil lawsuits relating air pollution health effects presented often times in Japan. As a evidence of causal relation between air pollution exposure and respiratory diseases, epidemiological studies were submitted to the courts. An interpretation of these studies by judges were markedly different and unique from ordinal epidemiological understanding; scientific understanding of the studies. Based on these unique implication of the evidence, the judgment announced forcefully recognizing a existence of causal relationship between exposure to pollution and disease outcome. The author wishes to let epidemiologists know the details of this phenomena, hoping a correct usage of epidemiology in the course of deciding judgment at civil lawsuit.

## INTRODUCTORY COMMENTS

During the period of Japan’s rapid, post-war economic growth, serious air pollution occurred in coastal, industrial zones. Among them, in the Yokkaichi area, asthma-like respiratory diseases were noted in patients in 1961. The Yokkaichi patients filed an anti-pollution lawsuit in September 1967. Subsequently, people in other newly established coastal, industrial areas and prewar, industrial districts filed lawsuits. Table 1 shows 8 lawsuits and describes the circumstances of each, their final status/resolution and the present situation.

**Table 1.** Overview of major law suite related to air pollution health effects.

case	year suite began	year of final resolution or present status	plaintiff	defendants	claims of plaintiff
Yokkaichi	1967	plaintiff won in the lower court, 1972	regional residens	industrial group	stop high pollutant levels, demand indemnity

Chiba	1975	plaintiff partly won, lower court, on appeal the case, settled, 1992	same	a steel company	same

Osaka	1978	same as above, 1998	same	indust.& road authori.	same as above,plus stop use of the road concrnd

Kawasaki	1982	same as above, 1999	same	same	same as above

Kurashiki	1983	same as above,1996	same	industrial group	stop high pollution levels, demand indemnity

Amagasaki	1988	plaintiff won in the lower court, 2000, on appeal the case settled	same	same	same as Kawasaki

Nagoya	1989	plaintiff partly won in lower court 2000, on appeal the case settled, 2001	same	same	same as above

Tokyo	1996	under consideration in lower court	same	same	same as above

To provide rapid relief to people exposed to air pollution, the Japanese Government enacted the Compensation Law for Pollution-Related Health Damage in Septemver 1973^1^^)^. Table 2 outlines the features of this law. Why this very specific law was passed was explained as the introduction of a kind of insurance system. It was to compensate not only people who had suffered from the pollution, but also to resolve setbacks in industrial activity as a result of frequent air pollution problems.

**Table 2.** Compensation law for pollution related health damage related health damage.

Based on absolute liability, concept as a supplement to the civil law system, to compensate sufferers more quickly		

Issued Sept. 1973, Revised March 1987		
I.	Substantial points of the law	
	1.	designated diseases: chronic bronchitis, pulmonary emphysema, bronchial asthma
	2.	designated areas: significant air pollution results in a wide outbreak of diseases: this means a symptom prevalence of 2-3times that of the normal rate, and pollution levels(SO2) exceeds 0.05ppm (annual average).
	3.	certification of patients a special committee organized by the local government considered designated materials of the patient who applied for certification as the designated patient

II.	Reasons of revision of the law and its state after revision	
	1.	because of decrease of levels of pollution, it became difficult to argue that designated diseases developed primarily because of exposure to pollution
	2.	after revision: cancellation of all designated areas no new recognition however, continued compensation for recognized patients

This article briefly describes problems associated with lawsuits over air pollution-related health damage and discusses issues concerning the understanding and implications of epidemiological studies that were submitted to courts as evidence explaining respiratory diseases in the plaintiffs.

## OVERVIEW OF THE LAWSUITS

All cases, except the first Yokkaichi trial, took many years to reach final resolution. Only the Yokkaichi trial ended in the lower court with a win by the plaintiffs; the defendants did not appeal. Table 1 shows the trial cases presented after the law issued includs the first Yokkaichi trial. After the Chiba case, plaintiffs became organized, with recognized patients and their claims were almost the same. -The levels of SO_2_ in the trial areas were presented, in Chiba and other areas, levels were lower than in Yokkaichi, so after the lower court judgment, the defendants appealed to the higher court.

Following consideration of the case by the higher court, the industrial defendants settled the cases and ended them by paying a settlement (judicial reconciliation). They said they had done so only from a business standpoint; they were in a hurry to end the lawsuits.

However, the road authority defendants did not join the settlements. Later, in 1998, the road authorities did settle with the plaintiffs and promised to cooperate in improving the environment around the road concerned, but they did not pay any settlement money in the Osaka trial. This was the first case where a settlement was reached between the road authorities and the plaintiffs.

Since then, cases have been resolved in a similar manner; Kawasaki’s trial ended in 1999 and very recently, the Amagasaki case was settled. In the Amagasaki case, even though the lower court recognized the plaintiffs’ claim to reduce traffic volume on the road concerned, the plaintiffs gave up their claim.

## WHY HAS THIS KIND OF TRIAL OCCURRED SO OFTEN?

1. General and common factors influenced the frequency of these trials. From the early 1970s, an anti-pollution movement prevailed nationwide and was especially strong in the designated areas. This movement continued until the late 1980s.

2. There were also local problems in specific regions (designated areas). Many people, especially those living in big cities, were active against pollution. They were ardent in organizing recognized people as plaintiffs. These movements had a political background. As a result, in Osaka, Kawasaki, and Amagasaki, trials occurred rather early.

3. Why was the Tokyo trial the latest? In Tokyo, automobile exhaust gases are the primary cause of air pollution and other sources are relatively minor. Also, people live in many different regions, with many people having traditional lifestyles. It was presumably difficult to organize those people in the same way as in another cities, where there was a more focused objective of anti-pollution action and/or preservation of the environment.

## USE OF EPIDEMIOLOGY IN THE TRIALS

In Japan, epidemiology is often used to presume a causal relationship in civil lawsuit in which it needs to decide causes of plaintiffs’ health damage (disease)^2^^)^. When the disease is unspecific nature, generally it is difficult in deciding the cause of the disease.

In this kind of decision making, judges depend on epidemiology for their proceeding to decide the cause of disease occurance. The principle of inference of epidemiologic causal relationship in lawsuit is not a specific way but ordinal method used in epidemiology. In addition to above epidemiologic causality, in case the causality can be infered with high probability, the causality becomes a judicial causal relation.

In judgment of air pollution lawsuits, always described a existance of judicial causality between plaintiff’s respiratory diseases and their exposure to air polution. Whether these decision are reasonable or not is the main problem of this article.

### 1.The cause of plaintiffs’ disease.

In every lawsuit, the plaintiffs always insisted that as they were the recognized patients described in the Compensation Law, their diseases (bronchial asthma, chronic bronchitis, emphysema, collectively referred to as COPD) were caused by exposure to air pollution.

However, the mechanism of recognition through the Law is clear-cut and simple; there is no medical diagnosis for each applicant. For the recognized patients, medical fees and compensation are provided every month, depending on the degree of their disease, as shown in Table 2. The plaintiffs also filed a civil lawsuit claiming money in addition to their compensation.

As a result in the court there was a major dispute as to the cause of their disease, that is whether or not their COPD was caused by air pollution.

Generally, it is a difficult matter to determine the cause in individual COPD patients because COPD is unspecific nature. As a consequence, the court in the dispute mentioned above did so based on epidemiological studies. As already noted in the first case of the Yokkaichi lawsuit, epidemiological studies conducted by a local medical college (Mie College of Medicine) showed a strong correlation between asthma incidence and ambient concentrations of SO_2_^3^^-^^5^^)^.

These studies were extensively examined in the court, as the plaintiff side’s key evidence and became an important basis for the plaintiffs’ win^6^^)^.

As shown in Table 1, the same kind of lawsuit was filed in various cities where high levels of ambient SO_2_ were reported. In most of these cities, epidemiological studies were conducted, investigating the relationship between the level of air pollution and the incidence of COPD observed among the inhabitants. These studies were submitted to the court as evidence by the plaintiffs.

### 2. An example of the evaluation and implications of epidemiological studies used in a trial.

In Yokkaichi city, the ambient SO_2_ level was so high that the 1 hour average concentration over 24 hours measured by the conduct metric analysis method often exceeded 5% over the 1 year measuring period^7^^)^. Given this, it was not a serious problem to ignore other air pollutants, and to treat SO_2_ as the key pollutant. Epidemiological studies in Yokkaichi were conducted under this unique pollution situation and among Yokkaichi plaintiffs there was not marked variety in their daily lives. As a result, it was not necessary to conduct a thorough exposure analysis in addition to the epidemiological studies done at that time in Yokkaichi^4^^, ^^5^^)^.

However, epidemiological studies submitted as evidence to courts after the Yokkaichi lawsuit, did not show clear relationships between air pollution and COPD symptoms among exposed people^8^^)^.

The principal reasons why these epidemiological studies did not show clear relationships are as follows: (1) in the cities in question, the pollution level was not as high as in Yokkaichi, (2) other pollutants, especially NO_2_ and SPM should not have been ignored, and (3) the lifestyles of the people were not so similar. Thus, a thorough exposure analysis was necessary.

In the late 1960s the ambient level of SO_2_ went down dramatically over the whole country^9^^, ^^10^^)^. After that, in the place of SO_2_, NO_2_ became the target pollutant. Sources of NO_2_ exist both in outdoors and indoors, so it is much more difficult to evaluate individual exposure levels than with SO_2_^11^^)^.

Such epidemiological studies without any satisfactory exposure analysis, could not lead to reasonable results. Next are presented two examples of lawsuit judgments, which concluded there was a causal relationship between plaintiffs’ COPD and air pollution exposure, based on unsatisfactory epidemiological studies.

### 1) The first trial in the Kawasaki lawsuit

Here, the lower court judgment in the Kawasaki lawsuit was announced collectively for the second, third, and fourth cases. The main feature of the judgment was that the importance of the pollutants that caused the plaintiffs’ COPD changed with time. When SO_2_ was talked about as the major pollution cause, the representative pollutant was SO_2_. When the levels of NO_2_ were properly measured and documented, was considered to be the representative pollutant that caused and aggravated respiratory problems. Attention later switched to SPM, which was found to be just as polluting as NO_2_^12^^)^.

With regard to the influence of atmospheric NO_2_, the Environmental Agency (at that time) presented numerous, detailed research finding^13^^)^. The findings from the research indicated that exposure to very low concentrations of NO_2_, that is below 30ppb, had no relationship to the onset of asthma or its aggravation^14^^)^. These findings came from sound and meticulous research; there were detailed evaluations of the exposures mentioned in the previous paragraph.

The court did not concern itself with evaluating these findings, and it did not take them into account in coming to its decision. Despite the high level of scientific reliability in these findings, they were not taken into account.

### 2) The first trial in the Amagasaki lawsuit

This judgment covered unusual ground and had no precedent. A ruling was made for the plaintiffs that the pollutant SPM caused COPD, based on a single epidemiological study^7^^)^. The basis of this ruling was study comparing asthma rates in three groups of children: those living along city roads, at some distance from roads, and in rural areas. The ambient levels of NO_2_ and SPM, the only measurable pollutants, were greatest along the roadside, lower some distance away from the road, and least in rural areas. The school children’s asthma prevalence decreased in the same way.

The authors of the study concluded that exposure to NO_2_ and SPM was related to the percentage of asthma development in school children^15^^)^. In the article written by those authors, more emphasis was placed on the NO_2_ value. This indicated that the actual measured value of NO_2_ was more abundant and substantial. The exposure analysis was also made using the data obtained. Despite this, the court ruled that the plaintiffs contracted COPD illnesses from exposure to SPM along the roadside.

In a lawsuit, the ruling will depend on whether past cases determining that the exposure to SPM would cause asthma and other COPD illnesses in the successive cases, epidemiological analyses were not considered in deciding the judgment. Also, school children were used in the epidemiological analysis; adult studies were not performed, even though the plaintiffs were all adults.

Two examples of trial judgments have been introduced above. In both cases, the judgments were made by an individual interpretation of epidemiological studies that there was a causal association between pollution exposure and COPD.

Besides the two examples of lawsuits introduced here, the interpretations used for judgments of causal relationships have basically been the same in other cases too. As a result, as shown in Table 1, except for Yokkaichi, in all the lawsuits the defendants have been defeated in the first trial. They appealed to the higher court and fought for reversal.

The reason for the lack of understanding of the epidemiological analyses among the judges’ interpretations was, as mentioned above, because it is difficult to evaluate the result of atmospheric pollution studies. I will explain later, but if one tries to make the direction and the contents of a judgment follow previous cases and the historical context, the tendency would be to go towards the idea that a causal relationship existed between atmospheric pollution exposure and COPD. The causal relationship will be found to exist no matter how conveniently the compensation law was created, as long as the patients need to be certified as “designated patients” under the law to receive money. As a result, difficult-to-evaluate epidemiological material will be applied forcefully to demonstrate a causal relationship. This partly explains the reason for making interpretations of epidemiological findings that are difficult to understand.

Regardless of the reason, this author believes that Japanese epidemiologists should take a close look at this situation, where judges interpret epidemiological findings in their own way to reach hard-to-swallow outcomes. In this material, we see differences between the scientific understanding and its evaluation in court rulings. It is the responsibility of the specialist, especially people who are interested in environmental epidemiology, to make this difference as small as possible^16^^)^.

## DISCUSSION AND SUMMARY

Again, examine the principle of an evidence ruling; based on epidemiological findings, the civil law will try to show a causal association between illness and environmental exposure. Various kinds of epidemiological findings have been presented as evidence, including studies on risk research and assessment. These studies were assessed and a legal ruling was made.

However, understanding risk research and risk management requires a high degree of specialized scientific knowledge. In court, the quality of evidence and its content will be evaluated, and this is the place where an objective evaluation should be made; it is not acceptable for the judges to make their own interpretation of the evidence. In a civil action, the purpose is to resolve the dispute between the parties^2^^)^. In order to make a conclusion for a continuous risk management and make a ruling, the judge has to depend on the scientific evaluations. Under these circumstances, the judge must take in consideration the contents of previous, similar rulings, and especially the historical background of pollutants and social problems. This is why a ruling in court is different from an objective scientific evaluation based on the material evidence.

I was motivated to publish this article because of a desire to introduce to epidemiologists all over the country to the unique epidemiological evaluation of atmospheric pollution, made in the ruling mentioned above. I believe, unlike in other countries, the frequent occurrence of many specific atmospheric pollution rulings in our country was mainly because our environmental pollution treatment was delayed and underdeveloped. Another reason that should not be forgotten is our political background, it can greatly influence a group to bring forward a lawsuit.

I sought to show the continuing importance of social events in civil actions, and how these studies have been used to those who deal with the specialized science of epidemiology. In any case, I wish to properly describe the dispute that has arisen because of the peculiar analysis in the judgment.
